# Feasibility randomised controlled trial comparing TRAK-ACL digital rehabilitation intervention plus treatment as usual versus treatment as usual for patients following anterior cruciate ligament reconstruction

**DOI:** 10.1136/bmjsem-2020-001002

**Published:** 2021-05-05

**Authors:** Emma Dunphy, Kate Button, Fiona Hamilton, Jodie Williams, Irena Spasic, Elizabeth Murray

**Affiliations:** 1 Research Department of Primary Care and Population Health, University College London, London, UK; 2 Physiotherapy Department, Homerton University Hospital NHS Foundation Trust, London, UK; 3 School of Healthcare Sciences, Cardiff University, Cardiff, UK; 4 Physiotherapy Department, Cardiff and Vale University Local Health Board, Cardiff, UK; 5 School of Computer Science and Informatics, Cardiff University, Cardiff, UK

**Keywords:** ACL, anterior cruciate ligament, physiotherapy, exercise rehabilitation

## Abstract

**Objectives:**

To evaluate the feasibility of trialling taxonomy for the rehabilitation of knee conditions—ACL (TRAK-ACL), a digital health intervention that provides health information, personalised exercise plans and remote clinical support combined with treatment as usual (TAU), for people following ACL reconstruction.

**Methods:**

The study design was a two-arm parallel randomised controlled trial (RCT). Eligible participants were English-speaking adults who had undergone ACL reconstruction within the last 12 weeks, had access to the internet and could provide informed consent. Recruitment took place at three sites in the UK. TRAK-ACL intervention was an interactive website informed by behaviour change technique combined with TAU. The comparator was TAU. Outcomes were: recruitment and retention; completeness of outcome measures at follow-up; fidelity of intervention delivery and engagement with the intervention. Individuals were randomised using a computer-generated random number sequence. Blinded assessors allocated groups and collected outcome measures.

**Results:**

Fifty-nine people were assessed for eligibility at two of the participating sites, and 51 were randomised; 26 were allocated to TRAK-ACL and 25 to TAU. Follow-up data were collected on 44 and 40 participants at 3 and 6 months, respectively. All outcome measures were completed fully at 6 months except the Client Service Receipt Inventory. Two patients in each arm did not receive the treatment they were randomised to. Engagement with TRAK-ACL intervention was a median of 5 logins (IQR 3–13 logins), over 18 weeks (SD 12.2 weeks).

**Conclusion:**

TRAK-ACL would be suitable for evaluation of effectiveness in a fully powered RCT.

**Key messages**

**What is already known**

- Rehabilitation following Anterior Cruciate Ligament Reconstruction can be lengthy and challenging.
- Digital health interventions may provide an opportunity to improve access to care and support self-management for people following Anterior Cruciate Ligament reconstruction.

**What are the new findings**

- Digital health tools such as the TRAK-ACL website, may provide an opportunity to teach and reinforce the key lessons and exercises at each stage of rehabilitation, as well as engage patients through behaviour change functions.
- DHI may be crucial for patients who do not have access to physiotherapy throughout rehabilitation.
- TRAK-ACL is a digital health intervention would be suitable for evaluation of effectiveness in a full powered RCT.

## Background

ACL injury is common in the active population and can require lengthy and challenging rehabilitation.^1–3^ Not all patients may have access to the physiotherapy care, information, education, exercise and knowledge needed at each stage of rehabilitation due to lack of time, experienced physiotherapists or specialist resources.^4 5^ It is reported that only 55% of individuals return to competitive sport and better outcomes are associated with individuals that complete at least 6 months supervised rehabilitation.^4 6 7^ However, only 30% of individuals with musculoskeletal conditions complete any rehabilitation beyond 6 months.^8^ It has been argued that digital health interventions (DHIs), such as websites and apps, may provide an opportunity to improve access to care and support self-management in areas where ACL rehabilitation may be inadequately resourced.^9^

There is a lack of evidence to support the use of DHIs for patient’s post-ACL reconstruction. One DHI which may be suitable for supporting patient’s post-ACL reconstruction is taxonomy for the rehabilitation of knee conditions—ACL (TRAK-ACL). TRAK-ACL stems from TRAK, an interactive for self-management support website,^10^ which is based on an ontology that describes standard care for the rehabilitation of knee conditions.^11^ TRAK-ACL focuses specifically on stage-by-stage rehabilitation after ACL reconstruction with the corresponding information presented using animations, videos, text and infographics. It includes a stage-by-stage exercise library and self-assessment criteria for progression. It was developed in line with the principles of the Behaviour Change Wheel, a framework for designing interventions and includes tools for self-monitoring and prompting engagement.^12^ Previous studies have indicated the acceptability of TRAK-ACL to both patients and clinicians as an adjunct to care.^13 14^

Given this preliminary evidence suggesting that TRAK-ACL may be acceptable to patients and physiotherapists, and could be integrated into routine National Health Service (NHS) care, it is appropriate to determine whether the intervention is an effective and cost-effective use of NHS resources. The Medical Research Council framework for developing and evaluating complex interventions highlights the importance of feasibility studies for testing procedures, estimating recruitment and retention and determining the sample size of a future randomised controlled trial (RCT).^15^ The process provides an opportunity to test the acceptability of recruitment pathways, outcome measures and uptake of the intervention to ultimately determine if a full-scale trial can be completed successfully.^16^ This paper details a randomised feasibility trial of TRAK for patients following ACL reconstruction which aimed to determine the feasibility of an RCT comparing TRAK-ACL plus treatment as usual to treatment as usual (TAU). Specific objectives were to: (1) assess the feasibility of recruiting and retaining participants to the RCT; (2) assess the feasibility of gathering costings data and patient reported outcomes; (3) assess implementation and fidelity issues such as participants’ and physiotherapists’ engagement with the website; (4) assess engagement with the mechanisms of behaviour change and (5) inform the protocol for a fully powered RCT to determine the clinical and cost-effectiveness of TRAK-ACL compared with TAU.

## Methods

### Design—a randomised controlled feasibility trial

This study was a parallel arm, individually randomised, feasibility RCT comparing postoperative ACL rehabilitation TAU with TAU plus TRAK-ACL. It is reported in line with the Consolidated Standards of Reporting Trials (CONSORT) reporting standards extension for pilot and feasibility trials.^17^ The trial was supported by the PRIMENT clinical trials unit, a registered UK Clinical Trials Collaboration and a trial steering committee was established to oversee the trial processes.

### Patient and public involvement (PPI)

This study won an award for patient involvement. TRAK-ACL was developed with a PPI group in a London NHS hospital.^13 18^ The group participated in choosing and developing content for the website, ensuring the website met an acceptable standard of diversity and inclusion and the design of the feasibility study. One member of the PPI group sat on the trial steering committee. The opinions of NHS Physiotherapists contributed to the development of the TRAK-ACL website and design of the feasibility study which was informed by the findings of two previous usability and acceptability studies carried out by this team of researchers.^13 14^

### Recruitment

The study took place at three NHS sites; a large University Hospital Foundation Trust, in an ethnically and socio-economically diverse English metropolitan area (site 1), a large South of England Trust covering a mixed urban and rural population (site 2) and large University Health Board in Wales, also mixed urban and rural population (site 3). Recruitment opened in July 2018 at sites 1 and 2. Site 2 was unable to recruit patients and was closed. Site 3 was added in November 2018 and recruitment closed in March 2019. Sites 1 and 3 are reported hereafter.

### Sample size

Feasibility study sample sizes are usually 50–70 participants.^19–21^ However, we looked to the ACL rehabilitation clinical trial literature for estimates of retention and compliance in similar studies.^22^ The literature suggested that we could expect between 20% and 25% loss to follow-up.^23 24^ We therefore estimated 75% retention. We estimated that 25 in each arm would give a 95% CI of 0.60 to 0.85, suggesting that we could be 95% certain that at least 60% of the target population would remain in the trial for at least 6 months.

### Randomisation

The trial statistician used computer-generated random number sequences to draw up a spread sheet where trial participant numbers could be added sequentially. Participants were allocated to the intervention or control arm by a member of the research team. Randomisation was performed after informed consent and baseline data were collected by the blinded assessor.

### Intervention

Participants randomised to the intervention arm received treatment as usual (TAU) plus TRAK-ACL website. The intervention was delivered by qualified chartered physiotherapists working in the musculoskeletal out-patient setting.

### TRAK-ACL website

TRAK-ACL is a DHI whose content was drawn from the literature on ACL rehabilitation. It was designed to support patients after ACL reconstruction by reinforcing teaching and exercise prescriptions given by a physiotherapist in face-to-face care. It includes an extensive exercise library and information from the ACL literature and physiotherapists and orthopaedic surgeons experienced in managing patients with ACL reconstruction. The exercises and information were provided phase by phase (early, middle, advanced and return to sport) as videos, animations, infographs and in written text format. Interactive features such as personal goal setting, progress logs and dashboards of progress were informed by the Behaviour Change Wheel framework for intervention design and are known to promote engagement with rehabilitation behaviours.^12^ The TRAK website can be accessed at: https://spas.cs.cf.ac.uk/trakacl/.

A training package was provided for physiotherapists using TRAK-ACL, including how to induct patients to TRAK-ACL. The TRAK-ACL intervention and the training programme are described in online supplemental file 1 and reported according to the Template for Intervention Description and Replication (TIDieR) guidelines.^25^ Each patient was given a TRAK-ACL alphanumeric login and they were invited to seek extra support with using TRAK-ACL if needed. Tablet computers were provided to participating sites and Wi-Fi availability was ensured before the study began.

10.1136/bmjsem-2020-001002.supp1 Supplementary data

The treating physiotherapist inducted the patient on how to use the TRAK-ACL website and give the participant a login during their first face-to-face consultation. The patient was shown a playlist of exercises which were individualised to their needs by the physiotherapist. Goals were set in discussion with the patient. Both goals and exercise playlists were progressed according to the patient reaching the rehabilitation milestones. All the rehabilitation exercises were on the TRAK-ACL website.

### Control group

Physiotherapy TAU varied across the included sites and is described in detail in online supplemental file 1. Common features included face-to-face time with physiotherapists, monitoring of the recovery from surgery and achievement of early rehabilitation goals. The duration of care was not restricted at either site and was expected to last for 6–12 months according to patient needs. A progression through phases of care was evident. The main difference between sites was the mode of delivery: at sites 1 and 2 care was delivered in an ACL group and at site 3 care was delivered though individual one-to-one appointments, with the option of attending a generalised lower limb class. The timing of appointments was different at each site. At sites 1 and 2 appointments were on a weekly basis dropping to fortnightly. At site 3 the scheduling was based on patient need and service capacity.

### Outcome measures

The primary outcomes were feasibility outcomes (recruitment and retention) plus measures to inform the parameters of a future trial.^26^ Study outcomes were taken at baseline, 3 and 6 months.

#### Primary study outcomes

- Feasibility of recruitment, measured by the number of people recruited to the trial (goal of four participants per month per site).
- Feasibility of retention, measured by the number of people still in the trial at the end of the study (goal of <30% drops-outs).
- Feasibility of collecting outcome measures, measured by the number of complete outcomes that were taken for each time point (goal of >80% at 6 months).
- Feasibility of collecting participants’ intervention usage data (goal of three or more logins per week per participant).
- Frequency of adverse events (goal <5%).

#### Secondary study outcomes

- Knee Injury & Osteoarthritis Outcome Score^27^ (KOOS): primary outcome of a future trial. This has five patient-rated scales to assess pain, symptoms, sport, activities of daily living and knee-related quality of life.
- Stanford Self-Efficacy Questionnaire; a patient-rated six-item questionnaire to evaluate self-efficacy of condition management in people with long-term conditions.^28^
- EQ-5D-5L, a patient-rated questionnaire to evaluate health-related quality of life.^29^
- The Client Services Receipt Inventory (CSRI) health economic tool, a questionnaire to evaluate health resource use and total costs incurred by patients, their employers, families and local healthcare services.
- Strength of quadriceps, which was measured using a standard gym leg press (kg).
- Calculation of sample size for a full trial.

The KOOS was chosen as the primary outcome of a future trial on patients with ACL reconstruction using a DHI because of its ability to measure both the impact of knee injury and longer term health outcomes such as osteoarthritis.^30^

### Data collection

Outcomes were collected at physiotherapy appointments at baseline, 3 and 6 months, except quadriceps strength, which was collected at site 1 at 3 and 6 months only. Outcome assessors and the lead researcher were blinded to treatment allocation but neither patients nor treating physiotherapists could be blinded as they needed to use the DHI.

### Data analysis

All primary outcome measures were summarised separately by study arm. Differences in secondary outcomes between arms were estimated using linear and logistic regression. Quantitative data were analysed as follows: binary and other categorical measures such as recruitment, retention and adverse events were summarised using frequencies and percentages. Continuous measures were summarised using means and SDs (or medians and IQRs for skewed distributions). The precision of estimates was assessed using 95% CIs. Power analyses were conducted to calculate the sample size necessary to detect an effect of the intervention in a future RCT with the KOOS as the primary outcome. Intention to treat principles were applied to data for all recruited patients.^31^ Progression of a full-scale trial would be considered if all of the feasibility criteria based on the primary outcomes were met.

## Results

### Recruitment

**Figure 1:**
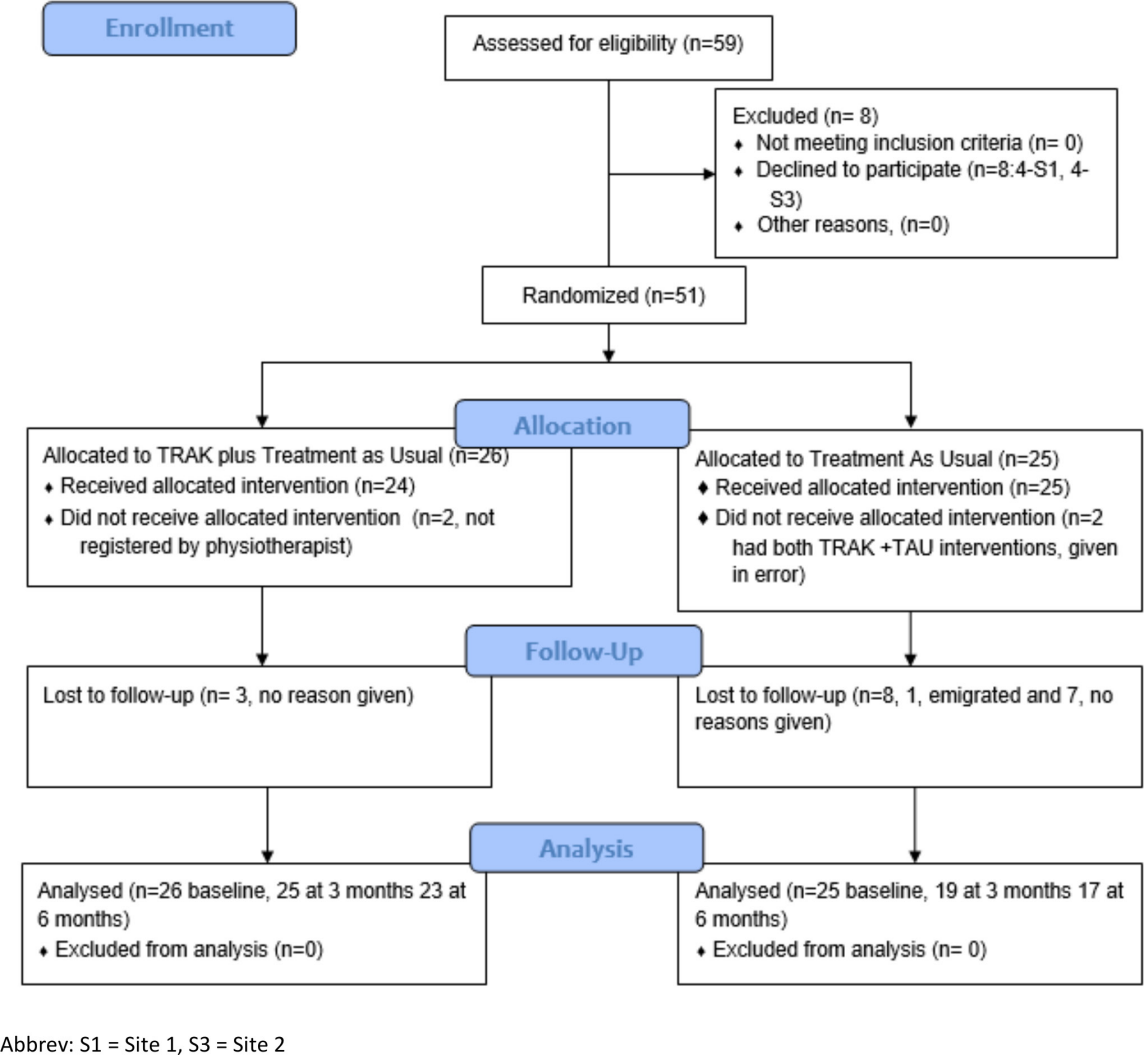
CONSORT flow diagram. TAU, treatment as usual; TRAK, taxonomy for the rehabilitation of knee conditions.

Flow of participants through the trial is illustrated using a CONSORT flow diagram shown in figure 1. Fifty-nine people were assessed for eligibility across two sites, of whom eight people declined to participate and 51 were randomised. Of these, 26 were allocated to TRAK-ACL and 25 to TAU.

### Characteristics at baseline

There were 51 study participants overall. The TAU arm and the TRAK-ACL arm were well-matched for baseline characteristics, presented in table 1.

**Table 1:** Baseline characteristics of participants

Patient characteristics	TAU	TRAK-ACL	Site 3	Site 1
	Mean (SD)	Mean (SD)	Mean (SD)	Mean (SD)
Age	28.4 (8.2)	30.8 (11.4)	29.0 (9.0)	29.82 (10.2)
Site	n25	n26	n10	n41
	**Freq (%)**	**Freq (%)**	**Freq (%)**	**Freq (%)**
Gender				
Female	12 (48.0)	11 (42.3)	3 (30.0)	20 (48.8)
Male	13 (52.0)	15 (57.7)	7 (70.0)	21 (51.2)
Ethnicity				
Asian	1 (4.0)	0 (0.0)	0 (0.0)	1 (2.4)
Asian other	0 (0.0)	1 (3.9)	0 (0.0)	1 (2.4)
Black	3 (12.0)	2 (7.7)	0 (0.0)	5 (12.2)
Mixed black and white	2 (8.0)	1 (3.9)	0 (0.0)	3 (7.3)
Mixed other	1 (4.0)	0 (0.0)	0 (0.0)	1 (2.4)
Mixed white and Asian	0 (0.0)	1 (3.9)	0 (0.0)	1 (2.4)
South Asian	0 (0.0)	2 (7.7)	0 (0.0)	2 (4.9)
White	18 (72.0)	19 (73.1)	10 (100)	27 (65.9)
Education level				
A level or equivalent*	3 (12.0)	5 (19.2)	1 (10.0)	7 (17.1)
Degree/higher degree	18 (72.0)	13 (50.0)	6 (60.0)	25 (61.0)
Diploma higher education	2 (2.0)	4 (15.4)	3 (30.0)	3 (7.3)
GCSE or equivalent†	2 (2.0)	4 (15.4)	0 (0.0)	6 (14.6)
Employment status				
Currently employed	20 (80.0)	19 (73.1)	7 (70.0)	32 (78.0)
Student	5 (20.0)	7 (26.9)	3 (30.0)	9 (22.0)

*A level: subject specific qualification for 16–18 years old.

†GCSE: subject specific qualification taken by 14–16 years old.

CONSORT, Consolidated Standard for Reporting Trials; GCSE, general certificate of seconday education; TAU, treatment as usual; TRAK-ACL, taxonomy for the rehabilitation of knee conditions—ACL.

#### Randomisation integrity

In TAU, two people were given the intervention by the treating physiotherapist by mistake. However, this was not known until the end of the feasibility study. Not all patients that were allocated to TRAK-ACL received the intervention. At site 3, two patients were allocated to the intervention but were never signed up to TRAK-ACL by the treating physiotherapist (table 2), although they continued to provide outcomes. At site 1, two patients were signed up to TRAK-ACL but never logged in. Hence usage data were only available for 22 participants from the TRAK-ACL group.

**Table 2:** Allocation to TRAK-ACL

TRAK-ACL usage	Total	Site 1	Site 3
	Freq (%)	Freq (%)	Freq (%)
Allocated to TRAK-ACL	26 (100)	21 (81.0)	5 (19.0)
Did not receive the intervention Reason	4 (15.0)	2 (8) Technical problems prevented login and unknown	2 (7.5) Not signed up by PT
Total for analysis	22 (85.0)	19 (73.0)	3 (11.5)
Received the intervention by error Excluded from analysis	n=2	n=2	n=0

PT, Physiotherapist; TRAK-ACL, taxonomy for the rehabilitation of knee conditions—ACL.

### Retention: numbers analysed

Retention was measured by the proportion of participants providing outcome data at 3 and 6 months. In total, 44 patients were retained to the study at 3 months and 40 at 6 months. Over the course of the study, three people were lost to follow-up in the TRAK-ACL arm and eight were lost to follow-up in TAU (table 3).

**Table 3:** Retention per treatment group and across sites

Retention	Total	TAU	TRAK-ACL
	Freq (%)	Freq (%)	Freq (%)
Retention			
Baseline	51 (100)	25 (100)	26 (100)
3 months	44 (86)	19 (76)	25 (96)
6 months	40 (78)	17 (68)	23 (88)
**Retention by site**		**Site 3**	**Site 1**
		**Freq (%)**	**Freq (%)**
Retention			
Baseline		10 (100)	41 (100)
3 months		7 (70)	37 (90)
6 months		7 (70)	33 (80)

TAU, treatment as usual; TRAK-ACL, taxonomy for the rehabilitation of knee conditions—ACL.

### Completeness of outcome data

The completeness of outcome data suggested that the number, complexity and time taken to complete items were all acceptable to participants, indicating that data collection for a fully powered RCT will be feasible. All but one outcome measure had 100% item completion for each outcome (table 4). The CSRI was the exception in that it was only completed by 54% of participants at baseline, and then 69% and 89% at 3 and 6 months.

**Table 4:** Outcome and data completeness

Outcome completeness	Total	TAU	TRAK-ACL
	Freq (%)	Freq (%)	Freq (%)
KOOS			
0	51 (100)	25 (100)	26 (100)
3	44 (86.27)	19 (76)	25 (96.15)
6	40 (83.96)	17 (68)	23 (88.46)
No of items	42	42	42
Complete items	42 (100)	42 (100)	42 (100)
Self-efficacy			
0	51 (100)	25 (100)	26 (100)
3	44 (86.27)	19 (76)	25 (96.15)
6	40 (78.43)	17 (68)	23 (88.46)
No of items	6	6	6
Complete items	6 (100)	6 (100)	6 (100)
CSRI			
0	51 (100)	25 (100)	26 (100)
3	44 (86.27)	19 (76)	25 (96.15)
6	40 (78.43)	17 (68)	23 (88.46)
No of items	114	114	114
Complete items	64 (54.38)	79 (69.29)	102 (89.47)
WPAI			
0	51 (100)	25 (100)	26 (100)
3	44 (86.27)	19 (76)	25 (96.15)
6	40 (78.43)	17 (68)	23 (31.08)
No of items	6	6	6
Complete items	6 (100)	6 (100)	6 (100)
EQ-5D-5L			
0	51 (100)	25 (100)	26 (100)
3	44 (86.27)	19 (76)	25 (96.15)
6	40 (78.43)	17 (68)	23 (31.08)
No of items	5	5	5
Complete items	5 (100)	5 (100)	5 (100)

Strength outcome	3 months	6 months	
Leg extension			
Total in study at site 1	37	33	
Yes	33 (89.18)	30 (90.90)	
No	4 (10.81)	3 (9.09)	
Reason	Pain or DNA	Pain or DNA	

Physiotherapy appointments	n=51, mean (SD)		
Total physiotherapy appointments per person	16.01 (5.04)		

EQ-5D-5L, an instrument for measuring generic health related quality of life

CSRI, Client Services Receipt Inventory; KOOS, Knee Injury and Osteoarthritis Outcome Score; TAU, treatment as usual; TRAK—ACL, taxonomy for the rehabilitation of knee conditions—ACL; WPAI, work productivity and impairment.

### TRAK-ACL usage data

**Figure 2:**
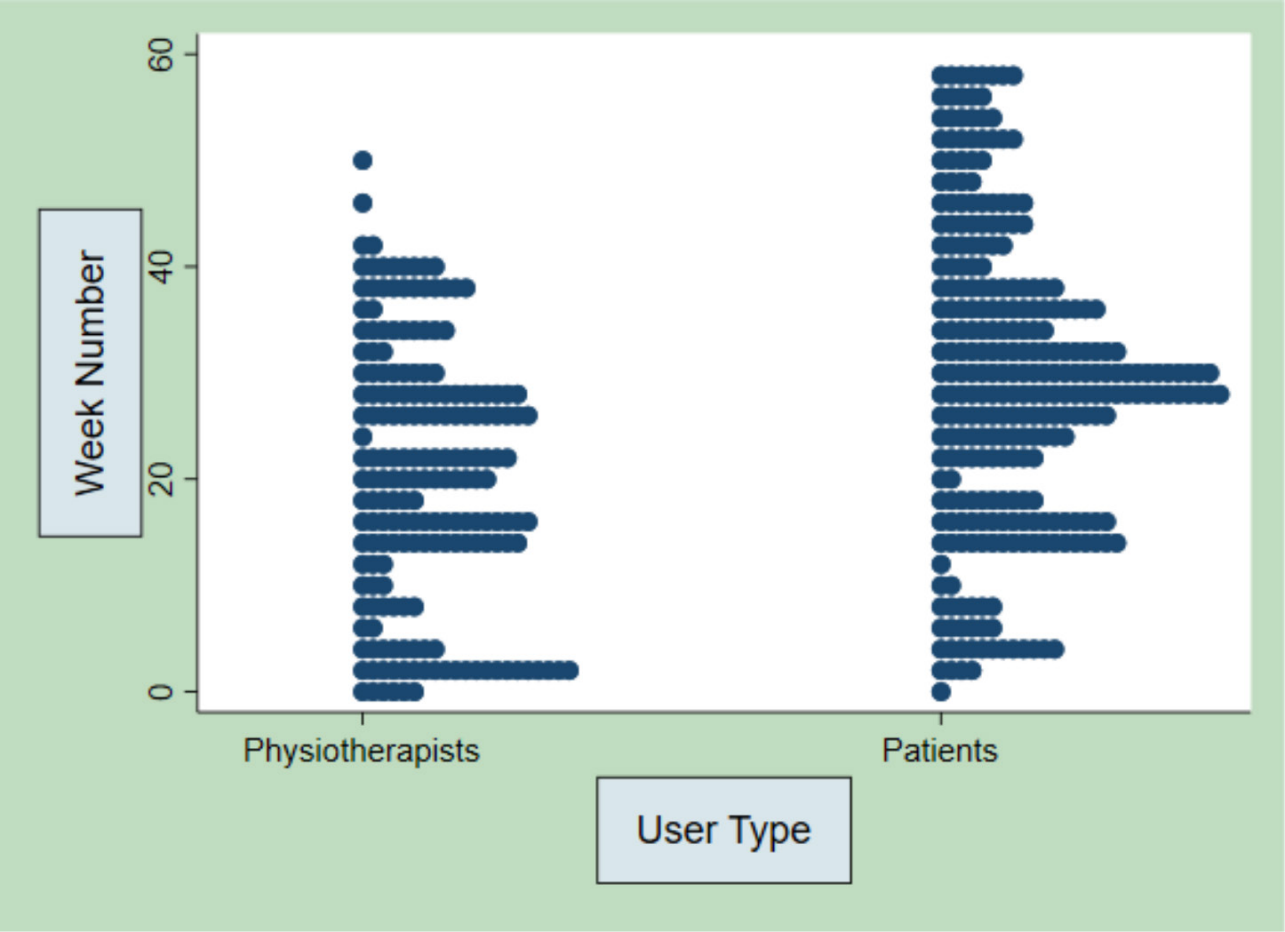
Physiotherapist and patient logins over 60-week duration of the feasibility trial.

Usage of the TRAK-ACL intervention was measured by the number of logins to the website, videos watched and behaviour change functions used, for example, exercise log, goal setting or weekly progress. These findings are displayed in full detail in online supplemental information 2—TRAK-ACL usage data. In summary, the median number of logins per patient participant was 5 with IQR of 3–13. The median (IQR) patient logins per week was 4 (2–7). The time between patients’ first and last login was a mean of 18 weeks (12.2SD), which suggests that users continue to engage over time and across phases of care. The median (range) physiotherapist logins per week was 2 (0–5). The median (range) logins per physiotherapist over the duration of the trial was 11.5 (6–18.5). The logins of physiotherapists and patients over 60 weeks, given in figure 2, showed consistency of patient usage as physiotherapist usage dwindles and disappears over the last 20 weeks.

10.1136/bmjsem-2020-001002.supp2 Supplementary data

There were no reported adverse events for participants enrolled on this feasibility trial.

### Analysis of secondary outcomes

The analysis of secondary outcomes is discussed in online supplemental information 3. The results show that the primary outcome of a future substantive RCT would be the KOOS at 6 months follow-up. A power calculation assuming a SD of 17.2671 on the KOOS estimates that a sample size of 172 participants (86 per study arm) will be required to detect a nine point difference on the KOOS between intervention and control groups with 90% power and 5% alpha. After inflation for 20% loss to follow-up, this figure increases to 108 participants per arm, 216 in total.

10.1136/bmjsem-2020-001002.supp3 Supplementary data

## Discussion

### Principal findings

The aim of the study was to determine the feasibility of an RCT comparing TRAK-ACL plus TAU to TAU in the management of postoperative patients with ACL. The findings suggest a future RCT to determine effectiveness, as measured by the KOOS, would be feasible, as patients were successfully recruited and retained and provided adequate outcome data, with the exception of the CSRI. Further work may be needed to improve data collection for an assessment of cost-effectiveness. The findings on usage suggest that patients and physiotherapists engaged with the TRAK-ACL intervention, in terms of number of log-ins, uptake of material and duration of engagement.

### Recruitment and retention

Eighty-six per cent of those approached about the study were recruited. The recruitment target of four patients per month was exceeded at site 1 where there was a dedicated researcher to facilitate recruitment. Site 2 recruitment was affected by changes to orthopaedic team leading to a significant drop in patients with ACL reconstruction. This could have been anticipated by better engagement with orthopaedics through the planning stage. It is known that recruitment challenges can be a key reason why randomised trials fail^32^ so a robust recruitment outcome was important.^33^ Recruitment method was highlighted as a key barrier to participation in previous DHI studies where in one example less than half of the physiotherapists allocated to delivering DHI actually recruited patients^34^ and in another only 30% of eligible patients were recruited.^35^ Strategies such as personalising the intervention (personal exercise plans) and dedicated staff support were implemented in the current study which may have contributed to recruitment success.^36^

At 6-month follow-up 78% of participants were retained, which exceed the criteria set for this study of less than 30% drop-outs. Eysenbach *et al* described two types of attrition; non-usage attrition (non-use of the DHI) and drop out attrition (lost to follow-up in the trial).^37^ Some patient loss can be explained by typical drop out from physiotherapy over the duration of ACL rehabilitation,^38^ and dropout rates can exceed 30% in trials for DHI and exercise.^14 39^ This study suggests that patients with ACL reconstruction will engage with the DHI and trial process over time and that the role of the research assistant seems key to facilitating outcome collection.

### Usage of TRAK-ACL

Usage data from TRAK-ACL show that there was consistent engagement by some users over time, indicating improved access to care. For the current study, a progression criterion of three logins per week per patient was set based on current evidence.^4^ Use of the behaviour change mechanisms such as logs, goal setting and educational and motivational content was accessed by up to 50% of patient TRAK-ACL users which may have improved these patients adherence to the target behaviour.

The usage data shown in this trial are similar to reported use data for health websites and apps used alongside treatment as usual.^35 40–42^ Additionally, levels of usage found in this study are similar to those found for other physiotherapy digital intervention feasibility studies^14 43^ and may indicate a growing acceptability of DHIs. A future trial would benefit from addressing physiotherapist engagement which may be affected by factors such as time and willingness to adopt new technology, understanding of new technology, the extent to which new technology meets user needs and the workplace support to maximise the potential of new technology.^34 44^

### Outcomes

The gathering of clinical outcomes such as KOOS, the self-efficacy score, EQ-5D-5L and work productivity and impairment was also shown to be feasible in this trial. There was 100% completion by all patients who were retained to the trial at 6 months in four out of five outcomes, which exceeded the feasibility progression criterion of 80% completion set for this study. The exception to good outcome collection was the CSRI. It is considered a valuable tool of health economic analysis and should be included where possible.^45 46^ In a future RCT, a digital rather than paper version could facilitate cleaner more usable data. Likewise, strength testing would be a key outcome of a future trial.^3 47^ The method of collecting strength testing across sites was not standardised and in a future trial isokinetic muscle strength testing of quadriceps and hamstrings at 180°/s should be considered. Feasibility of strength testing was successful however, clinical trial standard strength testing requires standardised conditions and calibration of machines.^48^

### Strengths and limitations

The strength of this study was the success of recruitment pathways, outcome measures and intervention toward determining feasibility. Progression to a full RCT would be recommended as the feasibility progression criteria for recruitment, retention, completeness of outcome measures at 6 months and adverse outcomes have all been met. The total sample size calculated for a full RCT was 216 participants (allowing for drop-outs). This would be achievable based on the recruitment to this study but would require 12 months’ recruitment over a minimum of five sites.

Not all patient participants engaged with all aspects of the behaviour change mechanisms built into TRAK-ACL and physiotherapists did not appear to sustain engagement with TRAK-ACL through to the return to sport phase of rehabilitation. Exploring this in a qualitative study would have strengthened this study and design of a future RCT. Measuring the number of face-to-face physiotherapist contacts would help evaluate if TRAK-ACL resulted in reduced physiotherapist contacts. The study was limited by the specificity of usage measurement that TRAK-ACL was capable of when considered against the AMUsED framework, a standard for reporting usage in digital trials.^49^ In a future trial, TRAK-ACL would need more specific usage measurement capabilities.

## Conclusions

The results of this study suggest that TRAK-ACL is suitable to go forward to a fully powered RCT investigating whether patients using a DHI as well as treatment as usual have better outcomes than patients receiving just treatment as usual. It is essential that sufficient support for trial delivery is built into a future RCT. Future research should aim to ensure that the DHI is stable and capable of measuring all relevant aspects of engagement before going to trial.

### New findings

Patient engagement with educational resources and exercises on the DHI occurs across multiple phases of the rehabilitation.

Digital health tools such as the TRAK-ACL website may provide an opportunity to teach and reinforce the key lessons and exercises at each stage of rehabilitation, as well as engage patients through behaviour change functions.

DHI may be crucial for patients who do not have access to physiotherapy throughout rehabilitation.

## Data Availability

Deidentified participant data are available upon reasonable request.
