# Supplementary material for: Feasibility randomised controlled trial comparing TRAK-ACL digital rehabilitation intervention plus treatment as usual versus treatment as usual for patients following anterior cruciate ligament reconstruction
Source: BMJ Open Sport Exerc Med. 2021 May 5;7(2):e001002. doi: 10.1136/bmjsem-2020-001002 (PMC8103946; doi:10.1136/bmjsem-2020-001002)
Supplement: Supplementary data [file bmjsem-2020-001002supp001.pdf]

**Supplementary Information 1 - The TRAK Website and Treatment as Usual**

# The Intervention

All participants received Treatment as Usual (TAU), which was delivered face-to-face and those in the intervention arm also received the TRAK-ACL website. TRAK-ACL has 3 views, one for participants, one for physiotherapists and a third administrator view.

### *TRAK-ACL website*

TRAK-ACL is an evidence based website specifically designed to support patients after ACL reconstruction. It reinforces the teaching and exercise prescription given in usual physiotherapy care. The website includes an extensive phase by phase (early, middle, advanced and return to sport) video exercise library. Physiotherapists can prescribe groups of exercises for their patients or guide them to work independently in a particular phase. It also includes a phase by phase library of evidence based information that is provided as animations, infographs, text and expert videos, to facilitate learning at each stage of care. Video participants included PPI participants and orthopaedic and physiotherapy colleagues who participated in the study. The website was developed in line with the Behaviour Change Wheel framework for intervention design(1) and it incorporated features such as educational materials, personal goal setting, progress logs and dashboards of progress which are associated with theories of behaviour change and can promote engagement with rehabilitation behaviours.

### *TRAK training for staff*

A two hour training event was provided at each site. It included the functionality of the TRAK-ACL website, the research process and the integration with current care. Training content was decided based on prior TRAK studies and feedback from previous users (2, 3).

Physiotherapist participants were informed about the research objectives, and the concepts of supported self-management and behaviour change that underpin the functions of TRAK-ACL. Each physiotherapist was taught to set up and manage a patient user and how to teach the patient to access and utilise the key functions of TRAK (Figure 1).

Further support was available through laminated guidance sheets that were on hand in the rehabilitation area to remind clinicians of the key points. Each physiotherapist participant had a summary of instructions emailed to them and further sessions of TRAK-ACL training were offered ad hoc for new starters or for anyone who requested support.

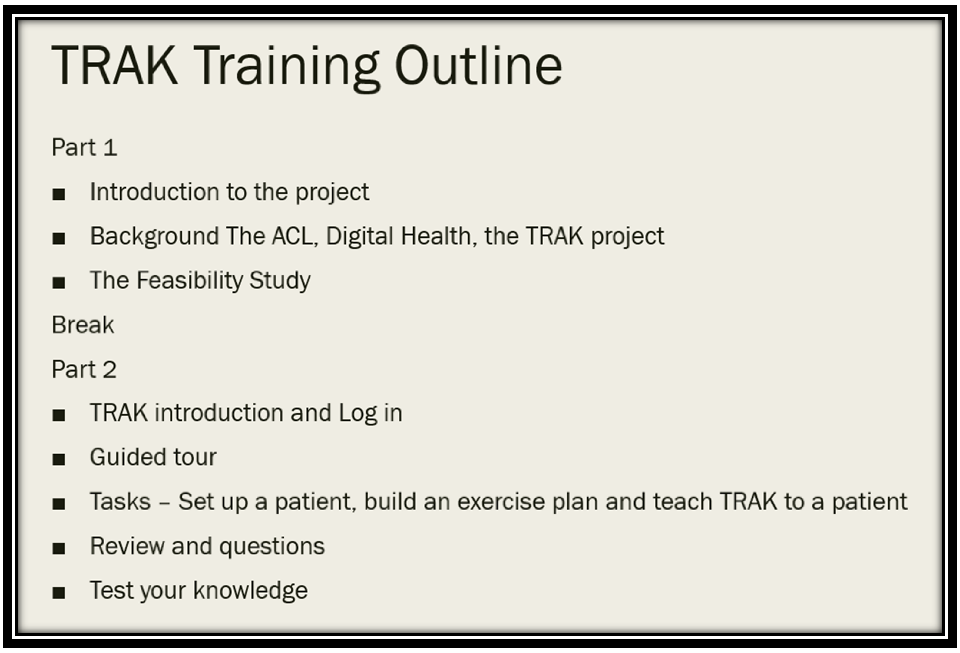


Figure 1. Contents of TRAK training

The results of a previous TRAK-ACL Acceptability study highlighted the need to make TRAK-ACL quicker for physiotherapists to use such as the inclusion of grouped exercise 'playlists' (2). Use of playlists were also included in training to facilitate a smoother integration of TRAK-ACL into usual clinical interactions. Figure 2 shows the functions where exercises could be selected as a group to save time, which we referred to as playlists, but the developer referred to as 'prescribe default exercises'. A further results of the previous study highlighted the need for efficient Wi-Fi and fast working tablets to use in a rehabilitation gym environment. The WiFi was checked at all sites and a MiFi (personal modem) was provided in one gym that did not have a good signal. Apple iPads were also provided, 2 at each site.

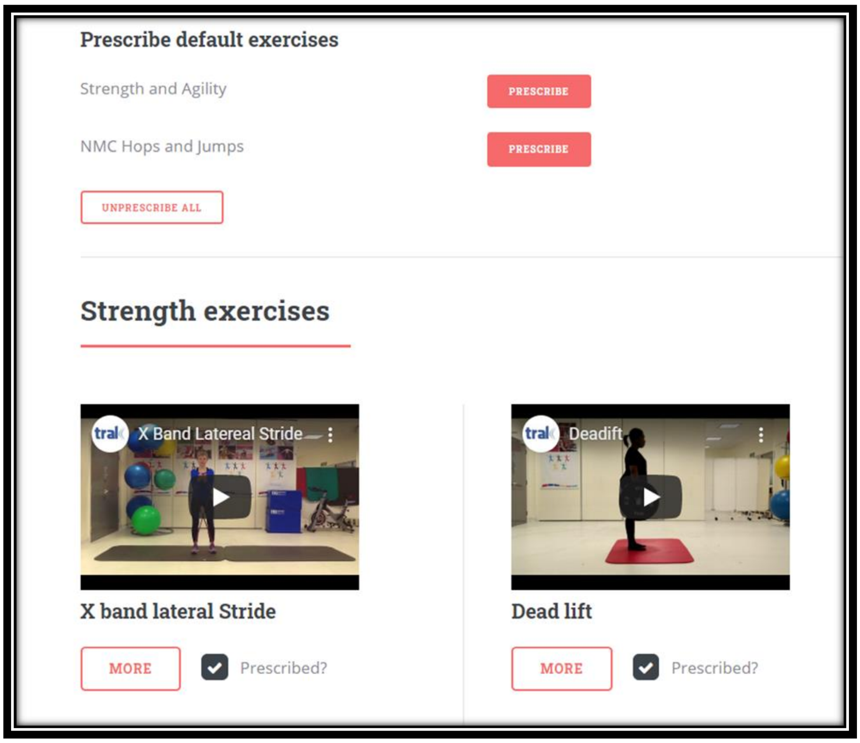


Figure 2. Playlists / 'Prescribe default exercises'

***Patient Participant Intervention Training***

Each patient in the intervention arm had a TRAK-ACL induction that was delivered by the treating physiotherapist. They were assigned an alphanumeric log in, which was a combination of their site location and the first four numbers of their birthdate. The password for all patients was set as 'patient'. Patients were guided to set goals, record progress and follow exercise plans as directed by their physiotherapist. Patients were invited to seek further TRAK-ACL support from their physiotherapist as needed.

## Treatment as usual

Treatment as usual for ACL rehabilitation was given to all study participants in both groups. The control group had treatment as usual only. Initial referral to physiotherapy always came from the orthopaedic department following ACL reconstruction. Following this the treatments varied by site.

***Treatment as usual at Site 1***

Patients were seen in an ACL group environment with separate classes for each phase. Care continued until rehabilitation goals were met and this could vary between individuals depending on

their goals but was usually from 6-12 months. Classes were one hour long. Initially they were weekly and they progressed to fortnightly once the patient achieved a set of criteria (usually between 12 and 16 weeks). Patient progression was based on meeting established criteria rather than time (4). They could choose to do a final return to sport phase depending on their personal goals. Attendance for this phase could be fortnightly or monthly depending on need.

Different time slots and classes accommodated the different phases of care and in each class the objectives of that phase were reinforced to the patients. Classes were well staffed so that individual assessment, problem solving, exercise prescription and management could occur.

**Treatment as usual at Site 2**

Treatment as usual at Site 2 had the same structure as Site 1. There were weekly dedicated ACL rehabilitation classes where patients progressed through phases of care. Unlike Site 1, Site 2 care was split across several sites.

***Treatment as usual at Site 3.***

At Site 3, patients were referred to the physiotherapy service and given an urgent post-operative priority. They had one-to-one physiotherapy at one of several sites across the catchment area. The initial appointment was 45 minutes and follow-up appointments were for 30 minutes. Induction was personalised but aimed to include reassurance, education, setting expectations, wound check, baseline outcomes and exercise education. Appointment frequency was determined by service capacity and needs of the patient based on physiotherapist assessment. When patients achieved an agreed criteria they could join a generalised lower limb rehabilitation class for 6 weeks, which was 1 hour long. They were reviewed in one-to-one care after this and had the option of being referred to a generalised advanced lower limb class which was held on a weekly basis. Continued attendance at the class was re-assessed after 6 weeks. Those who were unable to attend the exercise class continued to be managed on a one to one basis. The duration of treatment was based on patient need and there was no restriction on the duration of treatment.

***References***

1. Michie S, Van Stralen MM, West R. The behaviour change wheel: a new method for characterising and designing behaviour change interventions. Implementation science. 2011;6(1):42.
2. Dunphy E, Hamilton FL, Spasić I, Button K. Acceptability of a digital health intervention alongside physiotherapy to support patients following anterior cruciate ligament reconstruction. BMC musculoskeletal disorders. 2017;18(1):471.

3. Button K, Nicholas K, Busse M, Collins M, Spasić I. Integrating self-management support for knee injuries into routine clinical practice: TRAK intervention design and delivery. Musculoskeletal Science and Practice. 2018;33:53-60.
4. van Melick N, van Cingel RE, Brooijmans F, Neeter C, van Tienen T, Hullegie W, et al. Evidence-based clinical practice update: practice guidelines for anterior cruciate ligament rehabilitation based on a systematic review and multidisciplinary consensus. British journal of sports medicine. 2016;50(24):1506-15.
