# Supplementary material for: Feasibility randomised controlled trial comparing TRAK-ACL digital rehabilitation intervention plus treatment as usual versus treatment as usual for patients following anterior cruciate ligament reconstruction
Source: BMJ Open Sport Exerc Med. 2021 May 5;7(2):e001002. doi: 10.1136/bmjsem-2020-001002 (PMC8103946; doi:10.1136/bmjsem-2020-001002)
Supplement: Supplementary data [file bmjsem-2020-001002supp002.pdf]

Supplementary Information 2 – TRAK-ACL usage Data

This supplementary information presents the TRAK-ACL usage data that was collected to measure engagement with the various functions of TRAK-ACL.

Logins

Patient participants

There were a total of 279 patient logins over the 63 weeks of the study. There were 22 participants in total with one participant accounting for 113 logins. The median (interquartile range, IQR) number of logins from all patient participants per week was 4 (2-7). The median number of logins per participant was 5 IQR 3 – 13). The mean (Standard Deviation, SD) time elapsed between patients first and last log in was 18 (SD 12.2) weeks (Table 1). This suggests that patient users engaged with TRAK-ACL over time and different phases of care. The actual usage may be higher than reflected by the total number of logins as, unfortunately, logins were not timed out automatically and therefore patients could remain logged in over weeks and could use many TRAK-ACL features leaving no imprint.

Physiotherapists

Eight physiotherapists logged in 108 times over the trial. The median (range) physiotherapist logins per week was 2 (0-5) (Table 2). The median (range) logins per physiotherapist over the duration of the trial was 11.5 (6-18.5). The mean (SD) number of weeks of use was 28.8 (18.4). Figure 1 shows patient and physiotherapist logins over 20 weeks with the activity of the one patient 'superuser' separated out from other patient logins.

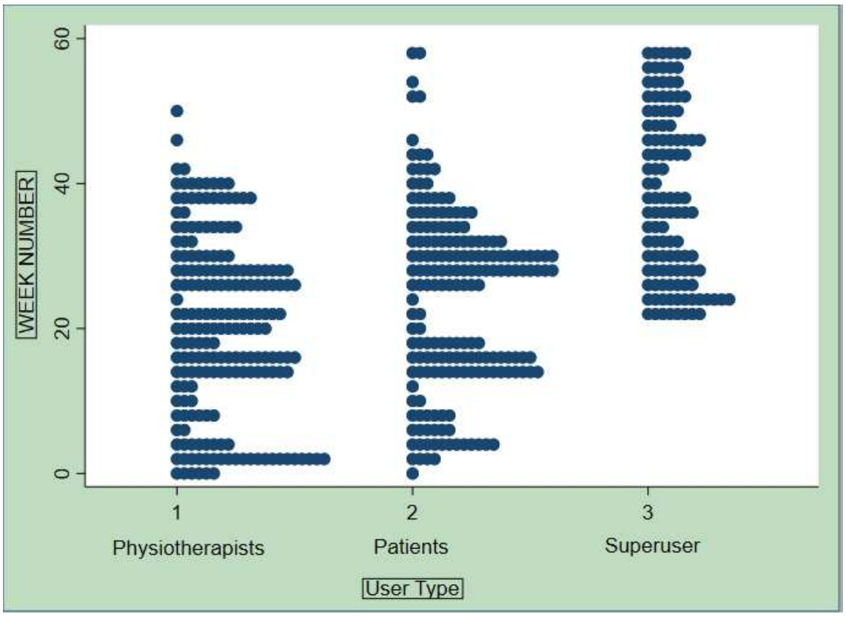

Figure 1. Physiotherapist and patient logins with 'superuser' separated over 20 week duration of the feasibility trial

Prescribed exercises

There were a total of 776 prescribed exercises. There was a median (IQR) of 34 (27-51) exercises prescribed per patient. The most commonly prescribed type of exercise was strength exercise with a total of 396 (51.03) strength exercises prescribed. Otherwise the exercise types were spread across a range of physical rehabilitation categories outlined in Table 1. The prescription of exercises was spread across the stages of care. Phase 1: Early Physiotherapy had 320 (41.24%). Phase 2: Intermediate Physiotherapy had slightly more at 373 (48.07%) while Phase 3: Advanced Physiotherapy had 83 (10.70%). There were no exercises prescribed in the Phase 4: Return to Sport Physiotherapy

TRAK Usage	
**Logins**	**Freq (%)**
Physiotherapist n=8	108 (27.90)
Patient n=22	279 (72.09)
	Median (IQR)
Logins per patient n=22	5 (2.0-17.0)
Logins per physiotherapist n=8	11.5 (6.0-18.5)
Logins p/week: Patient	4 (2.0-7.0)
Logins p/week : Physiotherapist	2 (0.0-5.0)
	Mean (SD)
Weeks of use (first to last log in) patients	18 (12.2)
Weeks of physiotherapist use	28.8 (18.4)
**Prescribed Exercises**	Median (IQR)
Prescribed exercises per patient	34 (27.0-51.0)
Prescribed exercises per physiotherapist (n=5)	163 (29.0-195.0)
Prescribed exercises per physio per week (n=5)	23.2 (15.0)
**Types of exercise prescribed**	**Freq (%)**
Total	776 (100)
Agility	37 (4.77)
Cardiovascular	13 (1.68)
Flexibility	108 (13.92)
Hops and Jumps	70 (9.02)
Neuromuscular Control	122 (15.72)
Run drills	8 (1.03)
Strength	396 (51.03)
Trunk Strength	22 (2.84)
**Exercise Phase**	**Freq (%)**
Phase 1: Early Physiotherapy	320 (41.24)
Phase 2: Intermediate Physiotherapy	373 (48.07)
Phase 3: Advanced Physiotherapy	83(10.70)
Phase 4: Return to Sport Physiotherapy	0

Table 1. Logins and Prescribed exercises

Behaviour change techniques	Median (IQR) n=22	Number of individual users	Number of uses	Spread
Goal Setting	1 (0-3)	11 users	43 Goals (n=11)	1-11 (n=11)
Weeks over which goals were used	4(4-8)			
Weekly Log	0 (0-1)	10 users	124 logs	1-71(n=10)
Weeks over which weekly log used	4 (4-12)			

Exercise Log	0 (0-16)	8 users	989 logs	3-713 (n=8)
Weeks over which exercise logs used	14 (8-20)			

Table 2. Behaviour Change Functions

Behaviour change functions

Table 2 described patients' use of behaviour change functions. There were 22 users of which 11 entered personal goals. These 11 entered a total of 43 goals, with a range of 1 – 11 goals per user. Of the 22 users, the median number of goals per user was 1 (IQR 0 – 3). Ten users utilized the weekly log function. They created 124 weekly logs with a spread of 1-71 logs per patient. The median weekly logs was 0 (0-1) n=22. The detailed exercise log had 8 users who entered 989 logs with a range of 3-713, and the median was 0 (IQR 0-16) n=22. This engagement of the 'superuser' appears to be responsible for the skew in results.

Educational videos and animations

TRAK itself was unable to measure videos clicked by user so the analysis of video usage was limited to YouTube's built in analytics. This allows views to be broken down into 'external' view sources which means sources other than a YouTube search, ie a link or implanted video on another website. Within these 'external sources', we could isolate the views that came directly from the TRAK-ACL website since they were labelled with the TRAK-ACL website server details. These videos are not available anywhere except TRAK-ACL so we can be sure that only study participants accessed these videos. Results are shown in Table 3.

The data is presented as number of views, average duration viewed as compared with total duration and percentage of overall video watched. The Phase 1 Animation was viewed 11 times (73% viewed). The Phase 2 Animation was viewed 11 times (82%). The Phase 3 Animation was viewed 7 times (87%) and the Phase 4 animation was viewed 6 times (78%). The Psychological factors of ACL rehabilitation animation was viewed 6 times (78%). These figures indicated an overall good uptake of educational material.

The expert videos proved less popular than the animations. The orthopedic surgeon expert video was viewed 7 times, for an average of 1 minute and 4 seconds (41%). The physiotherapist expert phase by phase videos were an alternative to the animations but with the same content. These were viewed less often, with the Phase 1 video viewed 4 times (59%). The Phase 2 video was viewed 2 times (12%), the Phase 3 video was not viewed at all. The Phase 4 video was viewed once for 21 seconds (8%). An expert video alternative to the psychological factors video was not viewed at all. A video demonstrating 'Good and Bad Knee Control' was viewed twice (100%). The combined uptake

of these videos and animations was good, however, since we cannot match them to individual users, we are unable to assess how many individual participants engaged with this educational material.

**Educational Videos**	**Views**	**Average View Duration**	**Total Duration**	**% of total viewed**
Orthopaedic Surgeon Video	7	00:01:04	4:02	26%
Phase 1 Animation	11	00:01:31	2:05	73%
Phase 2 Animation	11	00:02:20	2:50	82%
Phase 3 Animation	7	00:02:14	2:34	87%
Phase 4 Animation	6	00:02:10	2:46	78%
Psychological Factors Animation	6	00:01:59	2:32	78%
**Expert Videos**	**Views**	**Average View Duration**	**Total Duration**	**% of total viewed**
Expert video Overview of ACL rehabilitation	4	00:01:02	2:32	41%
Phase 1 Expert Video	4	00:01:26	2:26	59%
Phase 2 Expert Video	2	00:00:24	3:14	12%
Phase 3 Expert Video	0	0	4:15	0%
Phase 4 Expert Video	1	00:00:21	4:20	8%
Psychological Expert Video	0	0	4:14	0%
Good Control Bad Control	2	00:00:38	00:38	100%

Table 3. Educational videos and animations
