# Supplementary material for: Feasibility randomised controlled trial comparing TRAK-ACL digital rehabilitation intervention plus treatment as usual versus treatment as usual for patients following anterior cruciate ligament reconstruction
Source: BMJ Open Sport Exerc Med. 2021 May 5;7(2):e001002. doi: 10.1136/bmjsem-2020-001002 (PMC8103946; doi:10.1136/bmjsem-2020-001002)
Supplement: Supplementary data [file bmjsem-2020-001002supp003.pdf]

Supplementary information 3 - Analysis of secondary outcomes

KOOS

Each of the subscales of KOOS was analysed separately. KOOS Pain scores are shown in Table 1 for time point 0, 3 and 6 months. When comparing the two groups, TRAK-ACL and TAU, the intervention effect is measured by the p-value and 95% confidence intervals (95% CI). The mean difference in the intervention effect from baseline to 3 months for Pain was 3.97 (95% CI -1.803 to 9.756), p= 0.172 and 1.09 (-6.609 to 8.799), p=0.775, for baseline to 6 months. For KOOS Symptoms (Table 2) the intervention effect difference from baseline to 3 months was 1.49 (-5.603 to 8.596), p=0.673 and -6.70 (-17.096 to 3.687), p=0.199, for baseline to 6 months. For KOOS activities of daily living (ADL) the intervention effect difference between baseline to 3 months was 2.54 (-1.936 to 7.018), p=0.258 and 2.08 (-1.061 to 5.222), p=.188 for baseline to 6 months. For KOOS sport and recreation (SportRec) the intervention effect difference between baseline and 3 months was 7.8 (-6.51 to 22.111), p=0.278 and 2.27 (-10.007 to 14.560) ,p=0.71 for baseline to 6 months. Finally, for KOOS quality of life (QOL) the intervention effect difference between 0 and 3 months was 4.94 (-4.060 to 13.948), p=0.274 and -4.10 (-15.900 to 7.685), p=0.485 for 0 to 6 months. The KOOS scores at each time point for the 4 key KOOS domains are shown in Figure x. In all cases no difference was found between TRAK-ACL and TAU.

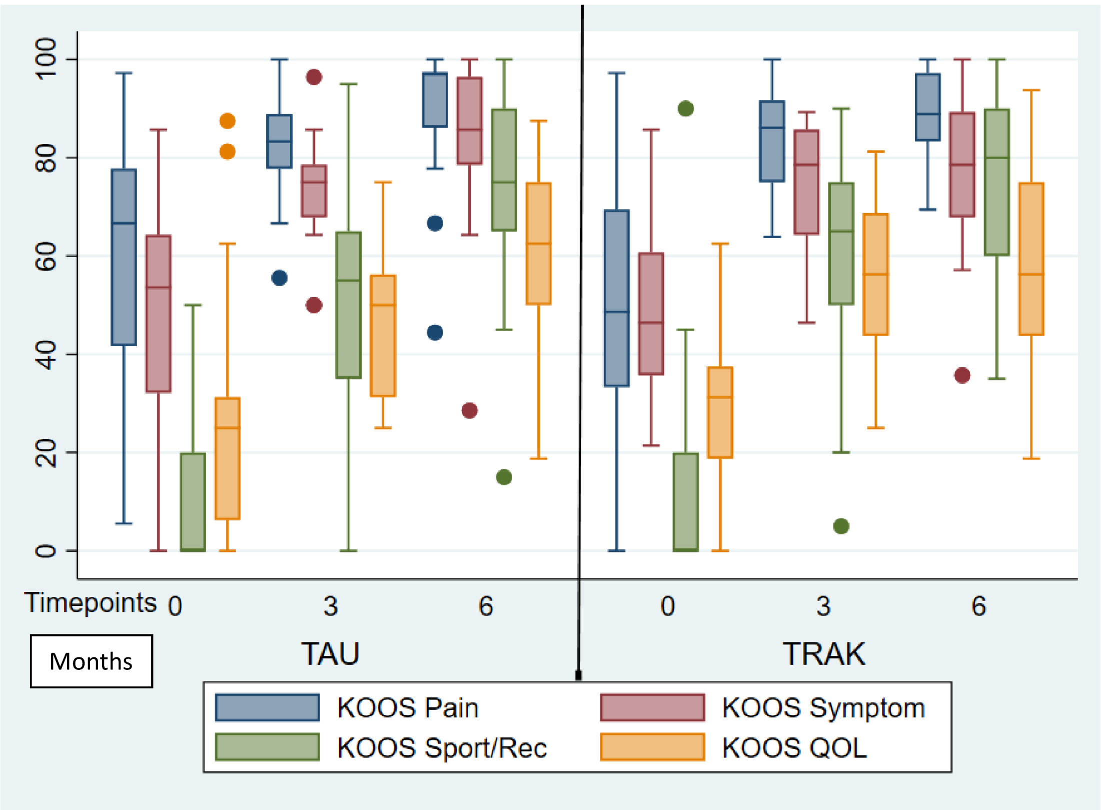

Figure x. Four domains of KOOS at 3 time points for TRAK and TAU.

Legend

TAU = Treatment as usual

TRAK= TRAK-ACL intervention arm

KOOS = Knee Osteoarthritis Outcome Score

QOL= Quality of Life

Outcome	TAU Summary Statistics N= , Mean (SD)	TRAK Summary Statistics N= , Mean (SD)	Intervention Effect Difference of means (95% CI) and p value
**KOOS**			
KOOS Pain			
0	25, 59.11 (24.00)	26, 51.28 (24.79)	
3	19, 82.31(10.68)	25, 84.22(10.10)	3.97 (-1.803, to 9.756) p= 0.172
6	17, 89.54(14.71)	23, 89.37(09.57)	1.09 (-6.609 to 8.799) p=0.775
KOOS Symptoms			
0	25, 49.71(12.17)	26, 47.12(17.02)	
3	19, 72.93(11.12)	25, 74.43(11.88)	1.49 (-5.603 to 8.596) p=0.673
6	17, 84.03(17.64)	23, 77.33(14.79)	-6.70 (-17.096 to 3.687) p=0.199
KOOS ADL			
0	25, 64.17(27.00)	26, 57.35(25.29)	
3	19, 91.87(08.06)	25, 94.17(06.40)	2.54 (-1.936 to 7.018) p=0.258
6	17, 95.76(06.13)	23, 97.44(03.61)	2.08 (-1.061 to 5.222) p=0.188
KOOS SportRec			
0	25, 9.8(14.10)	26, 10.58(20.51)	
3	19, 50 (23.51)	25, 57.8 (23.14)	7.80 (-6.51 to 22.111) p=0.278
6	17, 72.94(22.01)	23, 75.22(16.41)	2.27 (-10.007 to 14.560) p=0.71
KOOS QOL			
0	25, 25.25(24.70)	26, 28.37(17.87)	
3	19, 47.69(15.48)	25, 54.75(17.23)	4.94 (-4.060 to 13.948) p=0.274
6	17, 61.76(08.99)	23, 59.23(20.10)	-4.10(-15.900 to 7.685) p=0.485
**Self-Efficacy Score**			
0	25, 7.27(1.65)	26, 7.69(1.84)	
3	19, 8.30(1.39)	25, 8.43(1.00)	.126 (-0.601 to 0.854) p=0.728
6	17, 8.50(1.50)	23, 8.47(1.32)	-0.03 (-0.949 to 0.871) p= 0.932

Table 1. Exploration of Intervention effect data, KOOS and Self-Efficacy

KOOS: sample size calculation for a future trial

The developers of the instrument recommend that when using the KOOS, a change of 8-10 is considered the minimal clinically important difference and the standard deviation is set to 15 (14, 17, 19, 20).

Domain	Mean	SD
KOOS PAIN Baseline	55.11983	24.48464
KOOS Symptom Baseline	48.38936	20.10576
KOOS ADL Baseline	60.69781	26.11042
KOOS Sport/Rec Baseline	10.19608	17.49173
KOOS QOL Baseline	26.83824	21.33418

Mean KOOS5	= 40.24826	
Mean KOOS4	= 35.13588	
SD KOOS5		= 18.4658
SD KOOS4		= 17.2671

Table x. Means and SD for KOOS at baseline

The SD of 17.2671 from this feasibility trial compares reasonably to similar studies (20) (21) (22) (23). Therefore the primary outcome of a future substantive RCT would be the KOOS at 6 months follow up.

Self-Efficacy Score

The difference in the intervention effect from baseline to 3 months for the Self Efficacy score was .126 (-0.601, 0.854), p=0.728 and -.03 (-0.949 - 0.871), p= .932 for baseline to 6 months.
